# High incidence and remission of reported food hypersensitivity in Swedish children followed from 8 to 12 years of age – a population based cohort study

**DOI:** 10.1186/2045-7022-4-32

**Published:** 2014-10-13

**Authors:** Anna Winberg, Åsa Strinnholm, Linnea Hedman, Christina E West, Matthew S Perzanowski, Eva Rönmark

**Affiliations:** Department of Clinical Sciences, Pediatrics, UmeÅ University, UmeÅ, Sweden; Department of Public Health and Clinical Medicine, Occupational and Environmental Medicine, The OLIN Unit, UmeÅ University, UmeÅ, Sweden; Department of Environmental Health Sciences, Mailman School of Public Health, Columbia University, Columbia, USA

**Keywords:** Food hypersensitivity, Incidence, Remission, Risk factors, Sensitization

## Abstract

**Background:**

Few population-based cohort studies have examined reported food hypersensitivity longitudinally. We investigated prevalence, incidence and remission of perceived food hypersensitivity among schoolchildren from 8 to 12 years of age, and risk factors associated with incidence and remission.

**Methods:**

A population-based cohort including all 7–8 year-old children in three Swedish towns was recruited in 2006. A total of 2,585 (96% of invited) children participated in a parental questionnaire. The children in two of the towns, n = 1,700 (90% of invited) also participated in skin-prick-testing with airborne allergens. The cohort was followed using the same methods at 11–12 years of age. At study follow up, specific IgE to foods was analyzed in a randomized subset of children (n = 652).

**Results:**

The prevalence of perceived food hypersensitivity increased from 21% at 8 years to 26% at 12 years of age. During this four-year-period, the cumulative incidence of food hypersensitivity was high (15%), as was remission (33%). This pattern was particularly evident for hypersensitivity to cow´s milk, while the incidence of hypersensitivity to other foods was lower. Female sex, allergic heredity, current rhinitis and allergic sensitization were associated with the incidence of food hypersensitivity and allergic sensitization was negatively associated with remission. Risk-factor-patterns for both incidence and remission were different for hypersensitivity to milk compared with hypersensitivity to other foods. Generally, the agreement between reported food hypersensitivity and IgE-sensitization to the implicated food was poor.

**Conclusions:**

In this longitudinal, population-based cohort-study perceived food hypersensitivity was common among children between ages 8 and 12, often transient and not well correlated with food-specific IgE. While these findings suggest an overestimated prevalence of food hypersensitivity, the public-health-significance remains high as they reflect the perceived reality to which the children adapt their life and food intakes.

**Electronic supplementary material:**

The online version of this article (doi:10.1186/2045-7022-4-32) contains supplementary material, which is available to authorized users.

## Introduction

Food hypersensitivity (FHS) has emerged as a costly health problem in Western countries [1]. FHS is an umbrella term that includes reactions of both immunological (allergies) and non-immunological origin (intolerances) [2]. In a Swedish cohort-study, reported FHS affected 26% of children during the first 8 years of life, but the prevalence was halved when specific IgE and doctor´s diagnosis of food-allergy were required for classification [3]. The prevalence of both FHS and food allergy varies between studies due to differences in methodology, definition, age and ethnicity of the study populations. In two recent meta analyses investigating food allergy in Europe, the pooled life-time and point prevalence of reported food allergy were 17.9% and 5.9% respectively [4] and the lifetime challenge-proven prevalence of allergy to eight common foods varied between 0.1-0.6%. [5]. A meta-analysis including 51 studies investigating allergy towards 5 common foods showed that the prevalence of self-reported food allergy varied between 3-35% while challenge-proven food allergy varied between 1-11% [6]. As over-reporting of FHS is common [7, 8], thorough clinical diagnostics is essential in order to identify children with true FHS [1, 6]. Nevertheless, from a public health perspective it is important to have knowledge about the prevalence of perceived FHS, since experienced FHS can affect the child’s nutritional intake [9, 10] and has a negative impact on quality of life [11].

The natural course of FHS in schoolchildren is a research-area previously lacking of data [3, 12]. Few studies have investigated risk factors for FHS and most studies focus on IgE-mediated allergies [13, 14]. However, factors that have been associated with reported FHS are geographic setting [15, 16], age [3, 16], sex [17–19], eczema [3, 17], rhinitis [3, 17], asthma [3, 15] and allergic heredity [16]. This study was designed to investigate the incidence and remission of reported FHS and its associated risk factors in a Swedish population-based cohort followed from 8 to 12 years of age. We hypothesized that reported FHS would be common and that over this age-range, reported FHS to cow´s milk and hen´s egg would decrease while there would be an increase of reported symptoms to foods containing birch-pollen cross-reacting proteins.

## Materials and methods

### Study population

The pediatric cohort was established in 2006 as part of the OLIN-studies (Obstructive Lung disease In Northern Sweden) [20, 21]. All 2,704 children in first and second grade (age 7–8 years, median 8 year) in three towns in Northern Sweden were invited to a questionnaire-study and 2,585 (96%) participated. The 1,895 children in two of the towns (Kiruna and LuleÅ) were also invited to be skin-prick-tested (SPT) with ten common airborne allergens and 1,700 (90%) participated. In 2010 when the children were 11–12 years old (median 12 year), the cohort was followed using the same methods and 2,378 (89% of participants in 2006) answered the questionnaire. The participation rate was again very high in all areas. At the follow-up, all children in Kiruna and a random sample of children from LuleÅ were also invited to donate a blood-sample for analysis of IgE-antibodies and 652 (71%) children participated.

Both in 2006 and 2010 the studies were performed from February to April. The study was approved by the Regional Ethical Review Board in UmeÅ, Sweden.

### Questionnaires

The parental questionnaire included the International Study of Asthma and Allergies in Childhood (ISAAC) core questions [22] with added questions about symptoms, physician diagnosis, medication and possible determinants of asthma, rhinitis and eczema [20]. The questionnaire has been used since 1996 in a previous OLIN-cohort [20, 23]. For the current cohort the questionnaire was somewhat modified and questions regarding FHS were included (Additional file 1). The part of the questionnaire addressing FHS included the questions; “Has your child ever had an allergy/hypersensitivity to food” and “Does your child have an ongoing allergy/hypersensitivity to food”. Children reporting ongoing allergy/hypersensitivity also answered questions concerning the presence of reactions and type of symptoms to cow´s milk, hen´s egg, fish, wheat, soy, kiwi, orange, apple, raw carrots, banana, nuts, peanuts and almonds. The questionnaires were distributed by school-personnel and answered by the children’s parents.

### Skin prick test

The SPT followed the European Academy of Allergology and Clinical Immunology (EACCI) recommendations [24]. The panel included birch, timothy, mugwort, cat, dog, horse, two house dust mite extracts (Dermatophagoides pteronyssius and farinae) and two mold extracts (Claudosporium and Alternaria) (Solu-prick, ALK, Denmark). Inclusion of common allergenic foods to the SPT-panel was not possible due to safety reasons, since the SPTs were performed at the children’s schools. The potency of the extracts was 10 HEP except for the two molds, which were 1:20 w/v. Histamine 10 mg/ml and glycerol were used as the positive and negative controls. A positive test was defined as at least one wheal ≥3 mm in diameter, recorded after 15 minutes. The allergen potency and SPT-method were identical in the initial and follow-up study. The correlation between a positive SPT and specific IgE >0.35 kU/L to aeroallergens included in the test panel has been previously validated and was found to be excellent [25].

### Analysis of specific IgE

Food-specific IgE was measured in a random sample of children (n = 652) at study follow-up in 2010 at the age of 11–12 years. Specific IgE was measured in serum using Immuno-CAP (ThermoFisher Diagnostics, Uppsala, Sweden). The samples were initially analyzed using a food-screening Immune-CAP (fx5, ThermoFisher Diagnostics, Uppsala, Sweden), including cow´s milk, hen´s egg, cod, soy, wheat and peanut. If the screening test was positive (>0.35 kU/L,) specific IgE to all foods in the screening-test was analyzed separately. Specific IgE >0.35 kU/L was considered positive.

### Food hypersensitivity and other definitions

*Any FHS*: reported symptoms to at least one of the specific foods: cow´s milk, hen´s egg, fish, wheat, soy, kiwi, orange, apple, raw carrots, banana, nuts, peanuts and almonds. *Non*-*milk FHS* was defined as symptoms to one or more of the specific foods; hen´s egg, fish, wheat, soy, kiwi, orange, apple, raw carrots, banana, nuts, peanuts and almonds, cow´s milk excluded. *Milk FHS* was defined as reported symptoms to cow´s milk only (including symptoms of lactose intolerance). *Allergic heredity*: reported parental asthma, rhinitis and/or eczema. *Allergic sensitization*: at least one positive SPT. *Current asthma*, *rhinitis* and *eczema*: reported doctor´s diagnosis combined with use of medication or symptoms of disease during the last 12 months. Incidence of FHS was defined as absence of *any FHS* at 8 years of age and reported FHS to specific foods, *any FHS*, *non*-*milk FHS* and *milk FHS* respectively at 12 years of age. Remission of FHS was defined as reported symptoms of FHS to specific foods, *any FHS*, *non*-*milk FHS* and *milk FHS* respectively at 8 years of age and absence of the corresponding symptoms at 12 years of age.

### Statistical methods

Data were analyzed using the IBM SPSS Software version 21 (New York,, USA). The Chi-square test was used for comparison of proportions. Adjusted risk estimates expressed as odds ratio (OR) and confidence intervals were determined by logistic regression analysis for variables significantly associated to incidence or remission of FHS in the bivariate analyses. The independent variables included in the risk factor analyses were collected at age 8. The confidence interval was set to 95% (95% CI) and a p-value < 0.05 was considered statistically significant.

## Results

### Prevalence of FHS

**Figure 1:**
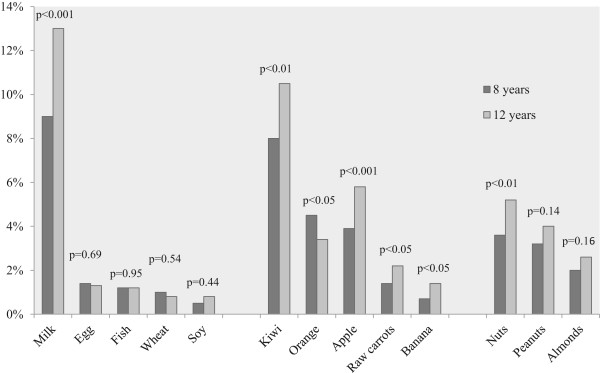
**Prevalence (%) of reported food hypersensitivity to specific foods at age 8 and 12 years.**

The prevalence of *any FHS* increased from 21.4% at 8 years to 25.9% at 12 years of age (p < 0.001). Changes in the prevalence of perceived FHS to each specific food are presented in Figure 1. The greatest increase was seen for cow´s milk, with a raise from 9% to 13% (p < 0.001). The prevalence of perceived FHS to hen´s egg, fish, wheat, soy, peanuts and almonds remained statistically unchanged with age.

### Incidence and remission

The incidence of *any FHS* from age 8 to 12 years was 14.7% and remission was 32.7% (Table 1). The specific food with the highest 4-year incidence was cow´s milk with 7.9% followed by kiwi with 4.6%, while the incidences were lower for other foods. The results were similar independent of whether children with no FHS or no FHS to the specific food at age 8 constituted the population at risk. Generally, remissions of FHS to all specific foods were high (27%-77%). Remission of *milk FHS* was 43.9%.

**Table 1:** **Cumulative incidence (%) and remission (%) of perceived food hypersensitivity (FHS) to different foods from age 8 to 12 years**

	Incidence (*)		Incidence (**)		Remission (***)	
FHS	n	(%)	n	(%)	n	(%)
Milk	147	(7.9)	190	(8.8)	94	(43.9)
Egg	8	(0.4)	11	(0.5)	15	(44.1)
Fish	4	(0.2)	10	(0.4)	12	(38.7)
Wheat	8	(0.4)	15	(0.6)	17	(77.3)
Soy	6	(0.3)	13	(0.5)	7	(58.3)
Kiwi	85	(4.6)	141	(6.5)	87	(44.6)
Orange	28	(1.5)	47	(2.1)	74	(69.2)
Apple	45	(2.4)	70	(3.1)	25	(26.9)
Raw carrots	9	(0.5)	37	(1.6)	17	(51.5)
Banana	6	(0.3)	26	(1.1)	8	(50.0)
Nuts	36	(1.9)	71	(3.1)	33	(38.8)
Peanuts	25	(1.3)	48	(2.1)	28	(36.8)
Almonds	16	(0.9)	40	(1.7)	25	(54.3)
*Any FHS*	274	(14.7)	274	(14.7)	167	(32.7)

*Children without any FHS at age 8 constituted the population at risk (n = 1868).

**Children without FHS to the specific food at age 8 constituted the populations at risk.

***Children with FHS to the specific food at age 8 constituted the populations at risk.

**Figure 2:**
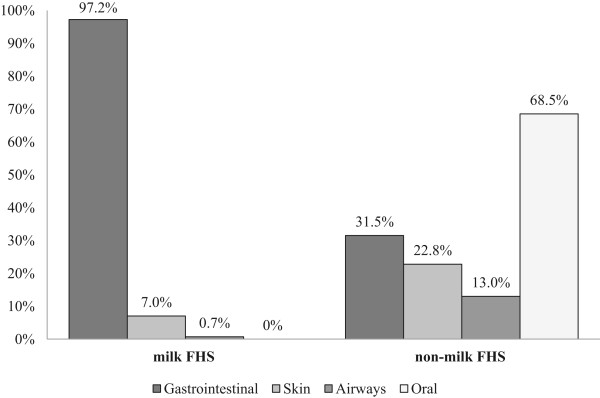
**Patterns of symptoms elicited by the offending foods among incident cases of food hypersensitivity,** ***milk FHS*** **(n = 162) and** ***non-milk FHS*** **(n = 143) respectively.**

The pattern of symptoms among incident cases of *milk FHS* differed significantly from incident cases of *non*-*milk FHS* (Figure 2). Therefore, *any FHS*, *non*-*milk FHS* and *milk FHS* were analyzed separately in the risk-factor-analyses.

### Factors associated with incidence

Baseline characteristics in the cohort at 8 years of age are presented in Table 2. In bivariate analyses, the incidence of *any FHS* during 4 years was significantly associated with female sex, allergic heredity, current asthma, current rhinitis and allergic sensitization. In the multivariate analysis female sex (OR 1.8 CI 1.3-2.5), allergic heredity (OR 1.6 CI 1.2-2.1), current rhinitis (OR 3.4 CI 1.9-5.9) and allergic sensitization (OR 1.6 CI 1.1-2.3) remained statistically significant. The variables associated with the incidence of *non*-*milk FHS* were similar to those associated with the incidence of *any FHS* in both the bivariate and multivariate analyses. The incidence of *milk FHS* was significantly associated with female sex (OR 1.7, CI 1.2-2.4) and allergic heredity (OR 1.6, CI 1.1-2.4) (Table 3).

**Table 2:** **Baseline characteristics at 8 years of age (%) for all children participating in the study and according to sex and living area**

	LuleÅ			PiteÅ			Kiruna			All		
	(n = 1242)			(n = 658)			(n = 478)			(n = 2378)		
Variables at 8 years	All	Girls	Boys	All	Girls	Boys	All	Girls	Boys	All	Girls	Boys
	(%)	(%)	(%)	(%)	(%)	(%)	(%)	(%)	(%)	(%)	(%)	(%)
Allergic heredity	57.6	56.1	59.1	63.5	61.7	65.0	57.5	54.0	60.9	59.2	57.1	61.2
Current asthma	4.5	3.2	5.8	7.1	5.0	8.9	9.2	6.8	11.5	6.2	4.4	7.8
Current rhinitis	5.8	5.7	5.9	6.2	4.4	7.8	6.7	5.1	8.2	6.1	5.2	6.9
Current eczema	11.4	11.3	11.6	10.0	11.1	9.2	11.5	9.8	13.2	11.1	10.9	11.2
Any positive SPT*	29.8	26.9	32.8	-	-	-	29.3	26.4	32.2	29.7	26.8	32.6

*The children from LuleÅ and Kiruna were invited to Skin prick testing.

**Table 3:** **Incidence (%) of** ***Any Food Hypersensitivity*** **(FHS),** ***non-milk FHS*** **and** ***milk FHS*** **in relation to risk factors and adjusted risk analyzed by multiple logistic regression analyses expressed as odds ratios (OR) with 95% confidence intervals (CI)**

		Incidence of ***Any FHS***			Incidence of ***non-milk FHS***			Incidence ***of milk FHS***		
Variables at 8 years		Adjusted risk*			Adjusted risk*			Adjusted risk*		
		(%)	P-value	OR (95% CI)	(%)	P-value	OR (95% CI)	(%)	P-value	OR (95% CI)
Sex	Boys	12.3		1.0	7.2		1.0	6.1		1.0
	Girls	16.9	0.005	1.8 (1.3-2.5)	10.4	0.012	1.8 (1.3-2.5)	9.5	0.006	1.7 (1.2-2.4)
Allergic heredity	No	11.0		1.0	6.3		1.0	5.7		1.0
	Yes	17.3	<0.001	1.6 (1.2-2.1)	10.6	0.001	1.6 (1.1-2.2)	9.2	0.005	1.6 (1.1-2.4)
Current asthma	No	14.0		1.0	8.1		1.0	7.5		
	Yes	23.9	0.010	1.2 (0.7-2.1)	19.3	<0.001	1.6 (0.8-2.9)	10.2	0.353	
Current rhinitis	No	13.5		1.0	7.8		1.0	7.6		
	Yes	40.3	<0.001	3.4 (1.9-5.9)	32.8	<0.001	4.0 (2.2-7.3)	10.4	0.359	
Any positive SPT	No	12.9		1.0	7.0		1.0	7.4		
	Yes	22.1	<0.001	1.6 (1.1-2.3)	16.3	<0.001	2.1 (1.4-3.2)	8.5	0.535	
Living area	LuleÅ	13.7			8.7			6.7		
	PiteÅ	13.1			7.6			7.9		
	Kiruna	18.2	0.067		10.3	0.362		9.7	0.154	

*Significant variables in the bivariate analyses were included in the multivariate model.

### Factors associated with remission

In bivariate analyses, remission of *any FHS* was negatively associated to allergic heredity, current asthma, current rhinitis and allergic sensitization. In multivariate analysis, only the association with allergic sensitization (OR 0.5 CI0.3-0.9) remained statistically significant. The variables associated with the remission of *non*-*milk FHS* were similar to those associated with remission of *any FHS* in the bivariate analyses. In multivariate analysis, current asthma (OR 0.5 CI 0.2-0.9) was the only variable significantly associated with *non*-*milk FHS*. The only variable significantly associated with remission of *milk FHS* was living in Kiruna, showing a negative association, OR 0.2 (CI 0.1-0.6) (Table 4).

**Table 4:** **Remission (%) of** ***Any Food Hypersensitivity*** **(FHS),** ***non-milk FHS*** **and** ***milk FHS*** **in relation to risk factors and adjusted risk analyzed by multiple logistic regression analyses expressed as odds ratios (OR) with 95% confidence intervals (CI)**

		Remission of any FHS			Remission of non-milk FHS			Remission of milk FHS		
Variables at 8 years		Adjusted risk*			Adjusted risk*			Adjusted risk*		
		(%)	P-value	OR (95% CI)	(%)	P-value	OR (95% CI)	(%)	P-value	OR (95% CI)
Female sex	Boys	32.7			31.8			48.2		
	Girls	32.8	0.994		36.4	0.348		43.4	0.488	
Allergic heredity	No	39.8		1.0	45.1		1.0	46.7		
	Yes	30.2	0.042	0.8 (0.5-1.1)	30.9	0.013	0.7 (0.4-1.1)	44.8	0.806	
Current asthma	No	34.6		1.0	37.2		1.0	46.2		
	Yes	18.6	0.014	0.5 (0.3-1.0)	17.9	0.005	0.5 (0.2-0.9)	33.3	0.333	
Current rhinitis	No	34.5		1.0	37.2		1.0	44.4		
	Yes	23.1	0.048	0.8 (0.4-1.4)	22.7	0.018	0.8 (0.4-1.4)	55.6	0.362	
Any positive SPT	No	37.9		1.0	45.0		1.0	42.7		
	Yes	22.4	0.001	0.5 (0.3-0.9)	22.9	<0.001	0.6 (0.3-1.0)	40.4	0.792	
Current eczema	No	34.5			37.7		1.0	43.9		
	Yes	27.2	0.128		25.5	0.024	0.8 (0.5-1.4)	51.2	0.390	
Living area	LuleÅ	33.9			36.5			46.7		1.0
	PiteÅ	35.9			33.7			56.0		1.6 (0.8-3.1)
	Kiruna	25.5	0.219		29.3	0.278		18.5	0.006	0.2 (0.1-0.5)

*Significant variables in the bivariate analyses were included in the multivariate model.

Several other risk factors were evaluated. None of the following variables were associated with neither incidence nor remission of any of the FHS-groups; having older siblings, living in house versus apartment, furred animals at home during the first 2 years of life, mother or father smoked, mother smoked during pregnancy and breastfeeding < 3 months.

### Correlation of Specific IgE and symptoms

The prevalence of a positive food-screening Immune-CAP was 17.0%. The prevalence of specific IgE to the foods included in the screening test; cow´s milk, hen´s egg, cod, soy, wheat and peanut, is presented in Table 5. In general, the agreement between the presence of symptoms and a positive food-specific IgE was poor. Only four out of 97 individuals reporting *milk FHS* were sensitized to milk and 40 out of 44 children with a positive specific IgE to cow´s milk did not report any milk-induced symptoms. In this subset of children, no one reported any symptoms to soy, though 16 were sensitized, and the 30 children with a positive IgE to wheat had no correlating symptoms. Of children reporting symptoms to peanut, 37% were peanut-sensitized and 38% of the children with a positive IgE-test for peanut described symptoms.

**Table 5:** Prevalence of specific IgE and reported symptoms to cow´s milk, hen´s egg, cod, soy, wheat or peanut and number of children with or without specific IgE > 0.35 kU/L and with or without reported FHS to the specific foods*

	Prevalence of spec IgE > 0.35 kU/L	Prevalence of reported FHS	Spec IgE > 0.35 kU/L and reported FHS	Spec IgE > 0.35 kU/L and no reported FHS	Specific IgE < 0.35 kU/L and reported FHS	Specific IgE < 0.35 kU/L and no reported FHS
	n=652	n=652	n	n	n	n
Cow´s milk	6.7%	14.9%	4	40	93	515
Hen´s egg	4.3%	2.2%	7	21	7	617
Cod	1.2%	1.8%	5	3	7	637
Soy	2.5%	0%	0	16	0	636
Wheat	4.6%	0.8%	0	30	5	617
Peanut	4.4%	5.7%	11	18	19	604

*Calculations based on the 652 children participating in measurement of specific IgE at age 12 years.

## Discussion

To our knowledge this is the first study to report data regarding incidence and remission of reported food hypersensitivity in a large population-based cohort of schoolchildren. We found an increase in the prevalence of perceived FHS from 21% at age 8 to 26% at age 12, with both high incidence and high remission. This pattern was particularly evident for FHS to cow´s milk. The high prevalence of any reported FHS was consistent with our hypothesis, while our predicted outcomes were contradicted by the high incidence of FHS to milk. Female sex, allergic heredity, current rhinitis and allergic sensitization were associated with the incidence of perceived FHS and allergic sensitization was negatively associated with remission. Incident cases of FHS to milk and non-milk foods were associated differently with symptoms and risk factors.

Female sex was a significant risk factor for the incidence of perceived FHS. Previous studies have shown a higher female prevalence of FHS among adolescents and adults [8, 17]. For food allergy, there is an increased risk among boys during childhood and a higher prevalence of food allergy among adult women [13, 19]. Endocrine influences have been suggested to contribute to this switch during adolescence [19]. In our cohort, reported FHS was more common among girls already at 8 years of age, mainly due to a higher female prevalence of FHS to cow´s milk [26]. This gender difference is in line with a recent Canadian study, where more women than men reported symptoms of lactose intolerance [27]. Also, symptoms of lactose malabsorption are associated with other diseases [10], of which irritable bowel syndrome is common even among younger people and has a female predominance [28]. However, since true lactose intolerance is reported to be rare among Swedish children [29] it is not likely to be a major contributor to the high prevalence of FHS in our study.

In the risk-factor-analyses, the incidence of *any FHS* and *non*-*milk FHS* during 4 years was significantly associated with current rhinitis and allergic sensitization. Furthermore, allergic sensitization was negatively associated with remission of *any FHS*. This is consistent with previous studies showing that atopy is a risk factor for incident and persistent FHS [3, 13, 14, 30]. Birch-pollen allergy and cross-reactions to birch-like proteins in vegetables are common in the Swedish population [31]. Others have shown that allergic reactions to fruits, nuts and peanuts constitute a large proportion of incident cases of FHS in Swedish schoolchildren [3]. This could explain the symptom-pattern among the *non*-*milk FHS*-cases, with a high percentage of symptoms of oral allergy syndrome (OAS) and rhinitis being the strongest risk factor associated with incidence of *non*-*milk FHS*.

The incidence and remission of *milk FHS* were differently associated with risk factors compared to incidence and remission of *non*-*milk FHS* and *any FHS*. Among incident cases of *milk FHS*, allergic heredity was the only atopy-related associated variable. It is difficult to find an explanation for this association. Since allergic heredity is a reported variable this could include reactions of non-IgE mediated origin. Also, parents with allergy might be more prone to suspect foods to cause symptoms in their children. The only variable associated with remission of *milk FHS* was living in the northernmost town, Kiruna, as compared to residence in other towns. This negative association could be a coincidence. However, the population in this area of Sweden includes inhabitants of Saami origin. The prevalence of lactose intolerance in Swedish Saami is unknown but among the Nenets, Samoyed people living in north-west Russia, the prevalence of the lactase down-regulating gene is high [32].

While food allergies are most common during childhood [6, 33, 34], the prevalence of reported FHS appears to remain high in adolescents and adults [4, 15–17]. In our study, there was a significant increase in the prevalence of perceived FHS from 8 to 12 years of age and this increase was mainly explained by a higher prevalence of reported FHS to milk among the 12-year olds. This finding could partly be explained by the onset of lactose intolerance [10]. Another feasible contributor could be an increased awareness in our society regarding foods, especially dairy products, as suspected triggers of various symptoms and diseases [10, 27, 28].

The high prevalence of a positive food-screening Immune-CAP supports the high prevalence of reported symptoms in our study population. However, the agreement between a positive IgE-test and reported symptoms was poor for all the tested foods. This was particularly evident for cow´s milk. A possible explanation for this finding could be parents reporting symptoms suspected to be induced by food in their children that either were not IgE-mediated or not actually caused by the suspected food. Also, IgE-tests can be false positive due to sensitization without symptoms, tolerance-development or cross-reactions [12, 31, 35]. Symptomatic FHS to soy and wheat are quite rare and these foods, and also peanut, have a high degree of cross-reactivity to birch- and timothy-pollens which could explain our findings. A Swedish study reported 7% of 8-year-olds to be sensitized to peanuts. The majority was sensitized to the birch-pollen-analog only and of these, only 17% experienced symptoms [31]. This could explain why only 38% of peanut-sensitized children experienced symptoms in the present study. However, since peanut allergy is mainly IgE-mediated [36], it is noteworthy that only 37% of children with reported FHS to peanut had a positive specific IgE.

A major strength of our study is the longitudinal and population-based design and the high participation-rate, supporting the representativeness of the population. Further, the children who contributed serum were very similar to the entire cohort with respect to the prevalence of allergic sensitization based on SPT, and indices of asthma and rhino conjunctivitis (data not shown). Furthermore, the prevalence of FHS in our study, although high, is consistent with previous findings [3, 6]. Studies investigating incidence and remission of FHS are rare and mostly performed on younger children or limited to one specific food [33, 34], which makes comparisons with our results difficult.

A possible study-limitation is parental reporting of symptoms, although a study from a previous pediatric OLIN-cohort showed a good agreement between reported symptoms of allergic diseases in teenagers and their parents [37]. Another study-limitation is that our results are mainly based upon reported data and since over-reporting of FHS is common [3, 6, 33], confirming studies using more objective diagnostic methods than specific IgE are needed. Nevertheless, previous studies show that people adapt their food-intakes according to perceived FHS, risking deficient intake of essential nutrients [9, 10]. Consequently, the high prevalence of FHS reflects the perceived reality to which children adapt their life and food-intakes.

## Conclusion

In summary, in this large population of schoolchildren followed over four years, there was a high prevalence, a high cumulative incidence and a high remission of perceived FHS. This pattern was particularly evident for FHS to cow´s milk, while the incidences of FHS to other specific foods were lower. Generally, there was a poor agreement between food-induced symptoms and IgE-sensitization to the implicated foods. Perceived FHS to milk and non-milk foods differed in risk factor patterns for both incidence and remission. Since one fourth of the children in this study reported FHS and since children adapt their life and food- intake according to perceived FHS, further studies including valid diagnostic methods are needed to evaluate the association between reported symptoms to food and true FHS. This is particularly important for the large number of children reporting FHS to essential foods like cow´s milk.

### Consent

Written informed consent was obtained from the patient’s guardian/parent/next of kin for the publication of this report and any accompanying images.

## Electronic supplementary material

Additional file 1: **The Olin Pediatric Questionnaire 2006.** (DOC 200 KB)

## Abbreviations

EAACI: European Academy of Allergology and Clinical Immunology
FHS: Food hypersensitivity
ISAAC: International Study of Asthma and Allergies in Childhood
OAS: Oral allergy syndrome
OLIN: The Obstructive Lung disease In Northern Sweden Studies
OR: Odds ratio
SPT: Skin prick test.
