# Supplementary material for: High incidence and remission of reported food hypersensitivity in Swedish children followed from 8 to 12 years of age – a population based cohort study
Source: Clin Transl Allergy. 2014 Oct 13;4:32. doi: 10.1186/2045-7022-4-32 (PMC4406030; doi:10.1186/2045-7022-4-32)
Supplement: Supplementary file 1 — Additional file 1: The Olin Pediatric Questionnaire 2006. (DOC 200 KB) [file 13601_2014_1065_MOESM1_ESM.doc]

**THE OLIN PEDIATRIC QUESTIONNAIRE 2006**

**Core questions – wheezing and whistling in the chest**

|  |  | YES | NO |
| --- | --- | --- | --- |
| **1.** | Has your child **ever** had wheezing or whistling in the |  |  |
|  | chest at any time in the past |  |  |
|  | If you answered “**No”,** please skip to question 6. |  |  |

========================================================================

|  |  | YES | NO |
| --- | --- | --- | --- |
| 2. | Has your child had wheezing or whistling in the chest |  |  |
|  | **in the last 12 months**? |  |  |
|  | If you answered “**No”,** please skip to question 6. |  |  |

_________________________________________________________________________________

|  |  |  | 1 to 3 | 4 to12 | More than |
| --- | --- | --- | --- | --- | --- |
|  |  | None | times | times | 12 times |
| 3. | How many attacks of wheezing |  |  |  |  |
|  | has your child had **in the last 12 months?** |  |  |  |  |

|  |  | Never woken with wheezing | Less than 1 night/ week | 1 eller1 or more nights/ week |
| --- | --- | --- | --- | --- |
| 4. | **In the last 12 months** how often, on average, has your child’s sleep been disturbed due to wheezing? |  |  |  |

|  |  | YES | NEJ |
| --- | --- | --- | --- |
| 5. | **In the last 12 months**, has wheezing ever been severe enough to limit your child’s speech to only one or two words at a time between breaths? |  |  |

========================================================================

|  |  | YES | NO |
| --- | --- | --- | --- |
|  |  |  |  |
| **6.** | Has your child **ever** had asthma? |  |  |
| **7.** | **In the last 12 months**, has your child’s chest sounded wheezy during or after exercise? |  |  |
| **8.** | **In the last 12 months**, has your child had a dry cough at night, apart from a cough associated with a cold or a chest infection? |  |  |

**Additional questions -** **wheezing and whistling in the chest**

|  |  | YES | NO |
| --- | --- | --- | --- |
| **9.** | **In the last 12 months**, has your child had wheezing or whistling in the chest without having a cold? |  |  |
| **10.** | In your opinion, does your child have the same physical fitness as friends of the same age? |  |  |
| **11.** | Does your child take **full part** in the physical education at school? |  |  |
| **12.** | **In the last 12 months**, has your child stayed home from school at any occasion due to breathing problems or asthma? |  |  |
| **13.** | Has your child been diagnosed by a physician as having asthma? |  |  |
| **14.** | Does your child see a physician on a regular basis for asthma? |  |  |

|  |  | Never | Some-times | Often/  Period-  ically | Every day |
| --- | --- | --- | --- | --- | --- |
| **15.** | How often has your child had to use asthma medications **in the last 12 months**? |  |  |  |  |

| **16.** | If your child had to use asthma medications, did he/she use any of the following? | YES | NO |
| --- | --- | --- | --- |
|  | Ventoline, Bricanyl, Inspiryl or other broncho-dilator |  |  |
|  | Becotide, Pulmicort or other corticosteroids |  |  |
|  | Lomudal or other |  |  |

|  |  | Did not have any problems/ Not at all | A little | A moderate amount | A lot |
| --- | --- | --- | --- | --- | --- |
| **17.** | In the last 12 months, how much did your child’s breathing problems/asthma interfere with his/hers daily activities? |  |  |  |  |
| **18.** | In your opinion, does your child’s breathing problems/asthma worsen at school? |  |  |  |  |

**Core questions – rhinitis**

|  |  | YES | NO |
| --- | --- | --- | --- |
| **19.** | Has your child **ever** had a problem with sneezing, or a runny, or a blocked nose when he/she **did not** have a cold? |  |  |
|  | If you answered “**No”,** please skip to question 25. |  |  |

====================================================================

|  |  | YES | NO |
| --- | --- | --- | --- |
| 20. | **In the last 12 months**, has your child had a problem with sneezing, or a runny, or a blocked nose when he/she **did not** have a cold? |  |  |
|  | If you answered “**No”,** please skip to question 25. |  |  |

____________________________________________________________________________

|  |  | |  | | | | | | YES | | NO | |
| --- | --- | --- | --- | --- | --- | --- | --- | --- | --- | --- | --- | --- |
|  | 21. | | **In the last 12 months**, has this nose problem been accompanied by itchy-watery eyes? | | | | | |  | |  | |
|  |  | |  | | | | | |  | |  | |
| 22. | | In which of the last 12 months did this nose problem occur? Please tick the boxes that apply | | | | | | | | | | |
|  | | January | |  | February |  | March |  | | April | |  |
|  | | May | |  | June |  | July |  | | August | |  |
|  | | September | |  | October |  | November |  | | December | |  |

|  |  | Not at all | A little | A moderate amount | A lot |
| --- | --- | --- | --- | --- | --- |
| 23. | **In the last 12 months**, how much did this nose problem interfere with your child’s daily activities? |  |  |  |  |

|  |  | Never | Some-times | Often/  Period-ically | Every day |
| --- | --- | --- | --- | --- | --- |
| 24. | How often has your child had to use medication for allergic nose/eye problems **in the last 12 months?** |  |  |  |  |

========================================================================

|  |  | YES | NO |
| --- | --- | --- | --- |
|  |  |  |  |
| **25.** | Has your child **ever** had “hay fever”? |  |  |

| **26.** | Has your child been diagnosed by a physician as having hay fever or allergic nose/eye problems? |  |  |
| --- | --- | --- | --- |

**Core questions – eczema**

|  |  | YES | NO |
| --- | --- | --- | --- |
| **27.** | Has your child **ever** had an itchy rash that was coming and going for at least six months? |  |  |
|  | If you answered “**No”** please skip to question 34. |  |  |

========================================================================

|  |  | YES | NO |
| --- | --- | --- | --- |
| 28. | Has your child had this itchy rash at any time **in the last 12 months**? |  |  |
|  | If you answered “**No”** please skip to question 34. |  |  |

|  |  | YES | NO |
| --- | --- | --- | --- |
| 29. | Has this itchy rash **at any time** affected any of the following places: the fold of the elbows, behind the knees, in front of the ankles, under the buttocks, or around the neck, ears or eyes? |  |  |

|  |  | Age <2 years | Age 2-4 years | Age >5 years |
| --- | --- | --- | --- | --- |
| 30. | At what age did this itchy rash first occur? |  |  |  |

|  |  | YES | NO |
| --- | --- | --- | --- |
| 31. | Has this rash cleared completely at any time **during the last 12 months?** |  |  |

|  |  | Never | Less than one night/ week | One or more nights/ week |
| --- | --- | --- | --- | --- |
| 32. | **In the last 12 months,** how often, on average, has your child been kept awake at night by this itchy rash? |  |  |  |

|  |  | Never | Sometimes | Often |
| --- | --- | --- | --- | --- |
| 33. | How often does your child use cortico-steroid ointment for his/her eczema? |  |  |  |

========================================================================

|  |  | YES | NO |
| --- | --- | --- | --- |
| **34.** | Has your child **ever** had eczema? |  |  |
| **35.** | Has your child been diagnosed by a physician as having eczema? |  |  |

|  |  | YES | NO |
| --- | --- | --- | --- |
| **36.** | Has your child ever had problems of nickel allergy, i.e. itch/rash from jewelry, e.g. necklaces; earrings, metal buttons, or buckles? |  |  |
| **37.** | Does your child have pierced ears? |  |  |

# Questions about food allergy/ food hypersensitivity

|  |  | JA | NEJ |
| --- | --- | --- | --- |
| **38.** | Does your child have an ongoing allergy/ hypersensitivity to any food?  **If your answer is yes – answer question 39.** |  |  |

| 39. | Does your child have symptoms caused by any of the following specific foods?  Please tick the boxes that apply. |
| --- | --- |

|  | Dont know | No symptoms | Itching of the mouth | Airway symptoms | Gastro-  Intestinal  symptoms | Skin symptoms |
| --- | --- | --- | --- | --- | --- | --- |
| Cow´s milk |  |  |  |  |  |  |
| Hen´s egg |  |  |  |  |  |  |
| Fish |  |  |  |  |  |  |
| Shellfish |  |  |  |  |  |  |
| Wheat |  |  |  |  |  |  |
| Soy |  |  |  |  |  |  |
|  |  |  |  |  |  |  |
| Apple |  |  |  |  |  |  |
| Peach |  |  |  |  |  |  |
| Kiwi |  |  |  |  |  |  |
| Avocado |  |  |  |  |  |  |
| Banana |  |  |  |  |  |  |
| Orange |  |  |  |  |  |  |
|  |  |  |  |  |  |  |
| Raw carrots |  |  |  |  |  |  |
| Potato |  |  |  |  |  |  |
|  |  |  |  |  |  |  |
| Peanuts |  |  |  |  |  |  |
| Nuts |  |  |  |  |  |  |
| Almond |  |  |  |  |  |  |
|  |  |  |  |  |  |  |
| Other? Specify |  |  |  |  |  |  |
| …………………….. |  |  |  |  |  |  |
| …………………….. |  |  |  |  |  |  |

**Your child’s background data**

| **40.** | What was your child’s birth weight?...................................grams |
| --- | --- |

| **41.** | Until what age was your child fed breast milk?...................................months |
| --- | --- |

| **42.** | At what age did your child first get milk supplementation/infant formula? ……................months |
| --- | --- |

| **43.** | How many siblings does your child have? ............................... |
| --- | --- |

| **44.** | As what number of the siblings was your child born? …....................... |
| --- | --- |

| **45.** | What is your child´s current length? ………………… cm |
| --- | --- |
| **46.** | What is you´r child´s current weight? ………………… kg |

| **47.** | Are there allergic problems present in other family members? Please tick the boxes which apply, even if the problems have disappeared. | Father | Mother | Sibling(s) |
| --- | --- | --- | --- | --- |
|  | Asthma |  |  |  |
|  | Allergic nose/eye problems |  |  |  |
|  | Eczema |  |  |  |
|  | Frequent bronchitis |  |  |  |

| **48.** | Did your child attend a day-care center before school age?  Please tick the appropriate option. | |
| --- | --- | --- |
|  | Never |  |
|  | First attended before the age of one year |  |
|  | First attended between ages 1 and 2 years |  |
|  | First attended after the age of 2 years |  |

| **49.** | Did your child attend family day-care before school age?  Please tick the appropriate option.. | |
| --- | --- | --- |
|  | Never |  |
|  | First attended before the age of one year |  |
|  | First attended between ages 1 and 2 years |  |
|  | First attended after the age of 2 years |  |

| **50.** | Has your child had: | YES | NO |
| --- | --- | --- | --- |
|  | Whooping cough |  |  |
|  | Croup |  |  |
|  | Pneumonia |  |  |
|  | Severe airway disease, e.g. RS Virus |  |  |
|  | Other severe infectious disease |  |  |

|  |  | YES | NO |
| --- | --- | --- | --- |
| **51.** | Does your child usually have colds more than 6 times per year? |  |  |

| **52.** | Does your child usually cough for more than 2 weeks when having a cold? |  |  |
| --- | --- | --- | --- |

**Your child’s home and environment**

**53.** Where and in what type of residence did your child live during his/her first year of life?

Please state the area of residence and tick the appropriate boxes regarding the type of setting (urban or rural) and the type of residence (house/terrace house or appartment). (If there were multiple residences, please state the one where the child spent the most time during his/her first year of life).

|  | Type of setting | | Type of resindence | |
| --- | --- | --- | --- | --- |
| Area of residence | Urban | Rural | House | Apartment |
|  |  |  |  |  |

**54.** Additional questions regarding your child’s current or previous home and environment.

Has any of the following occurred? Please tick the appropriate boxes in the table.

|  | Current residence | Previous residence | Never |
| --- | --- | --- | --- |
| Signs of damage from dampness or moulds |  |  |  |
| Occurrence of condensation/dampness on the inside of windows |  |  |  |
| Fire place/wood heating |  |  |  |
| A large busy road or a very frequented bus stop within 200 meters of the home |  |  |  |
| Car workshop, major garage or gas station within 200 meters of the home |  |  |  |
| Stable or barn within 200 meters of the home |  |  |  |

| **55.** | **Current type of residence:** | Year of construction | rooms, incl. kitchen | living area |
| --- | --- | --- | --- | --- |
|  | House/Terrace house |  |  |  |
|  | Apartment |  |  |  |

| **56.** | How many adults are living in the home? |
| --- | --- |
| **57**. | How many children are living in the home? |

**Animals and leisure time**

| **58.** | Do You presently keep or have You previously kept any pets during the time your child was growing up?  Please tick the applicable boxes in the table. | Presently | Previously | Never |
| --- | --- | --- | --- | --- |
|  | Cat |  |  |  |
|  | Dog |  |  |  |
|  | Rabbit/hamster/guinea pig |  |  |  |
|  | Other furred animal |  |  |  |
|  | Cage bird |  |  |  |
|  | Other pet |  |  |  |

|  |  | YES | NO |
| --- | --- | --- | --- |
| **59.** | Where there any furred animals in the home at any time period during your child’s first two years of life? |  |  |
| **60.** | Have you ever chosen **not** to keep pets (for example cat or dog) due to allergy in the family? |  |  |
| **61.** | Have you ever chosen **not** to keep pets (for example cat or dog) due to fear that your child might develop allergy? |  |  |

| **62.** |  | Presently | Previously | Never |
| --- | --- | --- | --- | --- |
|  | Does your family have or has previously had a farm? |  |  |  |

|  |  | YES | NO |
| --- | --- | --- | --- |
| **63.** | Does your child go horseback riding? |  |  |
|  | Does any other member of the family go horseback riding? |  |  |

|  |  | YES | NO |
| --- | --- | --- | --- |
| **64.** | Does your child regularly do indoor sports? |  |  |
|  | Does your child regularly do outdoor sports? |  |  |
|  | Does your child regularly do sports in an ice skating rink? |  |  |

| **65.** | **How frequently** does your child eat some kind of fruit? |  |
| --- | --- | --- |
|  | Every day, at least two |  |
|  | Every day, one in average |  |
|  | Almost every day |  |
|  | 1-3 times a week |  |
|  | Less than once a week |  |

| **66.** | **How frequently** does your child eat fish? |  |
| --- | --- | --- |
|  | At least three times a week |  |
|  | About twice a week |  |
|  | About once a week |  |
|  | About 1-3 times a month |  |
|  | Less than once a month |  |
|  | Never |  |

| **67.** | **How frequently** does your child eat fast food (i.e from Mac Donalds, Frasses, Max or other street food stands)? |  |
| --- | --- | --- |
|  | About once a day |  |
|  | About every other day |  |
|  | About twice a week |  |
|  | About once a week |  |
|  | A few times a month |  |
|  | Never/ Almost never |  |

**Rökvanor i familjen** Kryssa i tillämpliga rutor i tabellen.

|  |  | Does not smoke | Smokes  0-4 cig/day | Smokes 5-14 cig/day | Smokes 15-24 cig/day | Smokes  25 cig/day or more |
| --- | --- | --- | --- | --- | --- | --- |
| **68.** | Father |  |  |  |  |  |
|  | Mother |  |  |  |  |  |
|  | Other family member |  |  |  |  |  |

|  |  | No, never | Yes, at most once a week | Yes, >1 day per week |
| --- | --- | --- | --- | --- |
| **69.** | Does anyone smoke indoors,  or under the kitchen fan, in the home? |  |  |  |

|  | Did smoking occur at home during your child’s first year of life? | YES | NO |
| --- | --- | --- | --- |
| **70.** | Father smoked |  |  |
|  | Mother smoked |  |  |
|  | Other family member smoked |  |  |

|  |  | YES | NO |
| --- | --- | --- | --- |
| **71.** | Did the child’s mother smoke during the pregnancy? |  |  |
